# Sodium, Glucose and Dysregulated Glucagon Secretion: The Potential of Sodium Glucose Transporters

**DOI:** 10.3389/fphar.2022.837664

**Published:** 2022-02-14

**Authors:** Sarah L. Armour, Alexander Frueh, Jakob G. Knudsen

**Affiliations:** Section for Cell Biology and Physiology, Department of Biology, University of Copenhagen, Copenhagen, Denmark

**Keywords:** dapagliphlozin, SGLT1, SGLT2, metabolism, diabetes, Alpha cells

## Abstract

Diabetes is defined by hyperglycaemia due to progressive insulin resistance and compromised insulin release. In parallel, alpha cells develop dysregulation of glucagon secretion. Diabetic patients have insufficient glucagon secretion during hypoglycaemia and a lack of inhibition of glucagon secretion at higher blood glucose levels resulting in postprandial hyperglucagonaemia, which contributes to the development of hyperglycaemia. Sodium-glucose co-transporter 2 (SGLT2) inhibitors are an efficient pharmacologic approach for the treatment of hyperglycaemia in type 2 diabetes. While SGLT2 inhibitors aim at increasing glycosuria to decrease blood glucose levels, these inhibitors also increase circulating glucagon concentrations. Here, we review recent advances in our understanding of how SGLTs are involved in the regulation of glucagon secretion. Sodium plays an important role for alpha cell function, and a tight regulation of intracellular sodium levels is important for maintaining plasma membrane potential and intracellular pH. This involves the sodium-potassium pump, sodium-proton exchangers and SGLTs. While the expression of SGLT2 in alpha cells remains controversial, SGLT1 seems to play a central role for alpha cell function. Under hyperglycaemic conditions, SGLT1 mediated accumulation of sodium results in alpha cell dysregulation due to altered cellular acidification and ATP production. Taken together, this suggests that SGLT1 could be a promising, yet highly underappreciated drug target to restore alpha cell function and improve treatment of both type 1 and 2 diabetes.

## Main Text

Hyperglycaemia and insufficient release of insulin is characteristic for diabetes (American Diabetes Association., 2009). The obvious treatments to counter hyperglycaemia involve reduction of blood glucose by the administration of therapeutic insulin or other medication that reduces circulating glucose levels (American Diabetes Association., 2020). In addition to the changes in insulin action or release, secretion of the counter regulatory hormone glucagon from pancreatic alpha cells is also altered (Marliss et al., 1970; Unger & Cherrington, 2012). In healthy individuals, glucagon secretion is low during euglycaemia and circulating levels increase when blood glucose is reduced to stimulate hepatic glucose production (Walker et al., 2011; Finan et al., 2020). However, type 2 diabetic patients (T2D) are hyperglucagonaemic and this seems to contribute to the development of hyperglycaemia (Starke et al., 1987; Unger & Cherrington, 2012; Briant et al., 2016). Similar observations of hyperglucagonaemia have been described for type 1 diabetic (T1D) patients after ingestion of a mixed calorie meal (Bengtsen and Moller, 2021). In both type 1 and insulin dependent type 2 diabetic patients the role of glucagon, namely the response to hypoglycaemia is absent (Gerich et al., 1973; Cryer et al., 2003). This limits treatment possibilities substantially, since intensive therapeutic interventions via injection of insulin can result in severe and life threatening hypoglycaemia (Banarer et al., 2002). However, a lack of understanding of the mechanisms that control glucagon secretion has made it difficult to provide treatments that directly target the dysregulated glucagon secretion in both type 1 and type 2 diabetes.

### Paracrine Regulation of Glucagon Secretion

Alpha cells are under strong paracrine control by neighbouring beta and delta cells. At higher glucose concentrations, insulin and somatostatin act as strong inhibitors of glucagon secretion (Hauge-Evans et al., 2009; Kailey et al., 2012). This is part of the intra-islet communication hypothesis, suggesting that the dynamic crosstalk of high hormone levels found within the islet is the foundation of adequate secretion and regulation of insulin and glucagon (Banarer et al., 2002; Cryer, 2002; Hope et al., 2004; Meier et al., 2006). This could explain the dysregulated glucagon secretion in diabetic individuals as the intra-islet communication is compromised, with lack of beta cell function, elevated somatostatin secretion, and somatostatin resistance in alpha cells (Rorsman and Ashcroft, 2017; Kellard et al., 2020; Omar-Hmeadi et al., 2020). While this could explain hyperglucagonaemia, the mechanisms underlying the lack of glucagon secretion in response to hypoglycaemia remain unclear. Insulin deficient patients are treated with subcutaneous injection of therapeutic insulin, resulting in elevated insulin levels for at least 2 hours post injection (Home, 2012). The high levels of exogenous insulin in these patients could therefore lead to inhibition of glucagon secretion, in the absence of endogenous insulin (Cooperberg and Cryer, 2010; Yosten, 2018). Despite the paracrine influence on glucagon regulation, reductions in glucagon secretion from mouse and human islets already occur at glucose concentrations below 5 mM, where paracrine inhibition is absent, indicating that alpha cells also have an intrinsic mechanism that directly sense changes in circulating glucose levels.

### Intrinsic Glucose Sensing

**FIGURE 1:**
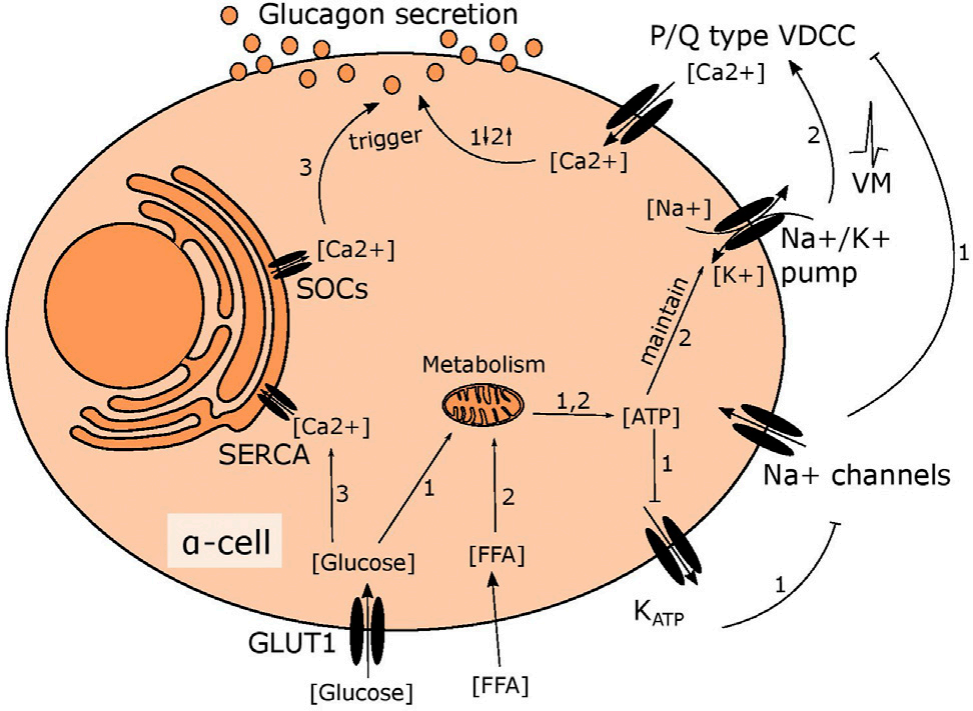
Suggested mechanisms of glucose induced changes in glucagon secretion. 1) In high glucose conditions, glucose is metabolised in mitochondria leading to higher ATP levels. This fully closes the K_ATP_ channel, depolarising the membrane slightly to inhibit the voltage gated sodium channel. This prevents opening of P/Q type VDCCs, limiting calcium influx and inhibiting glucagon secretion. 2) Fatty acid metabolism fuels the sodium potassium pump in low glucose, maintaining the membrane potential to allow action potential firing and activation of P/Q type VDCCs, resulting in stimulation of glucagon secretion 3) ER calcium stores are emptied in low glucose due to SOCs channel opening, triggering glucagon secretion. When glucose levels rise, this is reversed as the SERCA pump transports calcium from the cytosol into the ER.

At low glucose levels, alpha cells are thought to regulate glucagon secretion by intrinsic sensing of circulating glucose, but the exact mechanism is widely debated (Gromada et al., 2007; Walker et al., 2011; Gylfe, 2016). One hypothesis suggests that alpha cells are mirror images of beta cells. In beta cells, oxidation of glucose results in a rise of intracellular ATP levels proportional to extracellular glucose levels (Schuit et al., 1997). The increase in ATP leads to closure of the K_ATP_ channels and depolarisation of the plasma membrane. This activates voltage gated sodium channels, generating action potentials that trigger calcium mediated insulin release (Rorsman and Ashcroft, 2017). Observations such as the existence of similar channels in alpha cells, including the K_ATP_ channel, and calcium as the final trigger for glucagon release, led to the hypothesis that alpha cells sense glucose through a mechanism similar to beta cells (Figure 1) (MacDonald et al., 2007; Zhang et al., 2013). Contrary to this, it has been demonstrated that K_ATP_ channels in alpha cells are almost fully closed in very low glucose conditions (Liu et al., 2004; Zhang et al., 2013; Zhang et al., 2020) and suggested that the regulation of glucagon secretion relies on store operated calcium release (Figure 1) (Liu et al., 2004). Further, glucose oxidation in alpha cells is much lower than in beta cells, (Schuit et al., 1997) suggesting that increasing ATP levels as a readout for surrounding glucose availability is of little use for an alpha cell. Instead, alpha cells rely on fatty acids to fuel the response to hypoglycaemia in a mechanism that depends on the ATP dependent sodium-potassium pump rather than the K_ATP_ channel (Figure 1) (Briant et al., 2018). The importance of the sodium-potassium pump for basal secretion, suggests that maintenance of intracellular sodium is important for alpha cell function and in line with this, sodium glucose transporters (SGLT)s have recently been suggested to play a central role for glucagon secretion (Bonner et al., 2015; Muhlemann et al., 2018; Knudsen et al., 2019; Suga et al., 2019).

### Sodium Glucose Transporters and the Unresolved Issue of Expression

The role of SGLT1 and 2 in glucose homeostasis and diabetes is well established in regard to glucose reabsorption (Freitas et al., 2008; Hummel et al., 2011; R.A. DeFronzo et al., 2012; Gorboulev et al., 2012). Here, SGLT2 is a low affinity transporter, highly expressed in the proximal renal tubule where it plays a major role in glucose reabsorption, accounting for up to 90% of glucose reabsorption from the kidney (R.A. DeFronzo et al., 2012). In contrast, SGLT1 is a high affinity glucose transporter and although SGLT1 also plays a role in glucose reabsorption in the kidney (R.A. DeFronzo et al., 2012), it primarily functions in the small intestine where it is pivotal for intestinal reabsorption of glucose (Gorboulev et al., 2012). The contribution of SGLT 1 and 2 to pancreatic islet function and the control of gluco-regulatory hormone release is still debated (Bonner et al., 2015; Suga et al., 2019; Chae et al., 2020). Several functional studies have attempted to determine the presence of SGLT2 in islets using SGLT2 inhibitors, a class of prescription medicine, which target both SGLT2 and SGLT1, albeit the latter with much lower affinity. SGLT2 inhibitors improve glycaemia in glucose intolerant patients by increasing glycosuria (Cefalu et al., 2015a; Cefalu et al., 2015b; Zinman et al., 2015). However, patients taking these inhibitors often present with higher circulating glucagon (Ferrannini et al., 2014; Merovci et al., 2014). Similar findings in rodent studies using the inhibitor dapagliflozin showed that inhibition of SGLT2 increased glucagon secretion at higher glucose concentrations (Bonner et al., 2015; Pedersen et al., 2016). Contrary to this, others have found that perfusing rat pancreas with dapagliflozin or phlorizin, had no effect on glucagon, insulin or somatostatin secretion (Kuhre et al., 2019). One potential source of the discrepancies between studies is the specificity and pharmacology of the SGLT2 inhibitors. Whilst all SGLT2 inhibitors bind preferentially to SGLT2, the selectivity for SGLT2 over SGLT1 varies considerably from ∼1.5-fold for phlorizin, to ∼2,500-fold for Empagliflozin (Cinti et al., 2017).

At the transcriptional level, both SGLT1 and SGLT2 have been detected in human islets (Bonner et al., 2015; Mawla and Huising, 2019) however, even here there is some disagreement as other studies have been unable to detect mRNA or protein expression of SGLT2 in rodent and human islet cells (Timper et al., 2016; Kuhre et al., 2019; Suga et al., 2019; Chae et al., 2020). The discrepancies in reports of SGLT2 expression in pancreatic islets have been suggested to be caused by inter-individual heterogeneity of SGLT2 expression (Saponaro et al., 2020). While the studies exploring the role of SGLT2 are inconclusive, the presence of SGLT1 has been more consistently detected in islet cells (Schuit et al., 1997; Timper et al., 2016; Knudsen et al., 2019; Suga et al., 2019; Chae et al., 2020), with several studies demonstrating enrichment of SGLT1*,* but not SGLT2, mRNA in human alpha cells and at the protein level in mouse alpha cells (Timper et al., 2016; Suga et al., 2019). Collectively, this suggests that pancreatic islets express greater levels of SGLT1 than SGLT2, and that SGLT1 expression may be specific for alpha cells.

### Glucose Transport, SGLTs and the Inhibition of Glucagon Secretion

**FIGURE 2:**
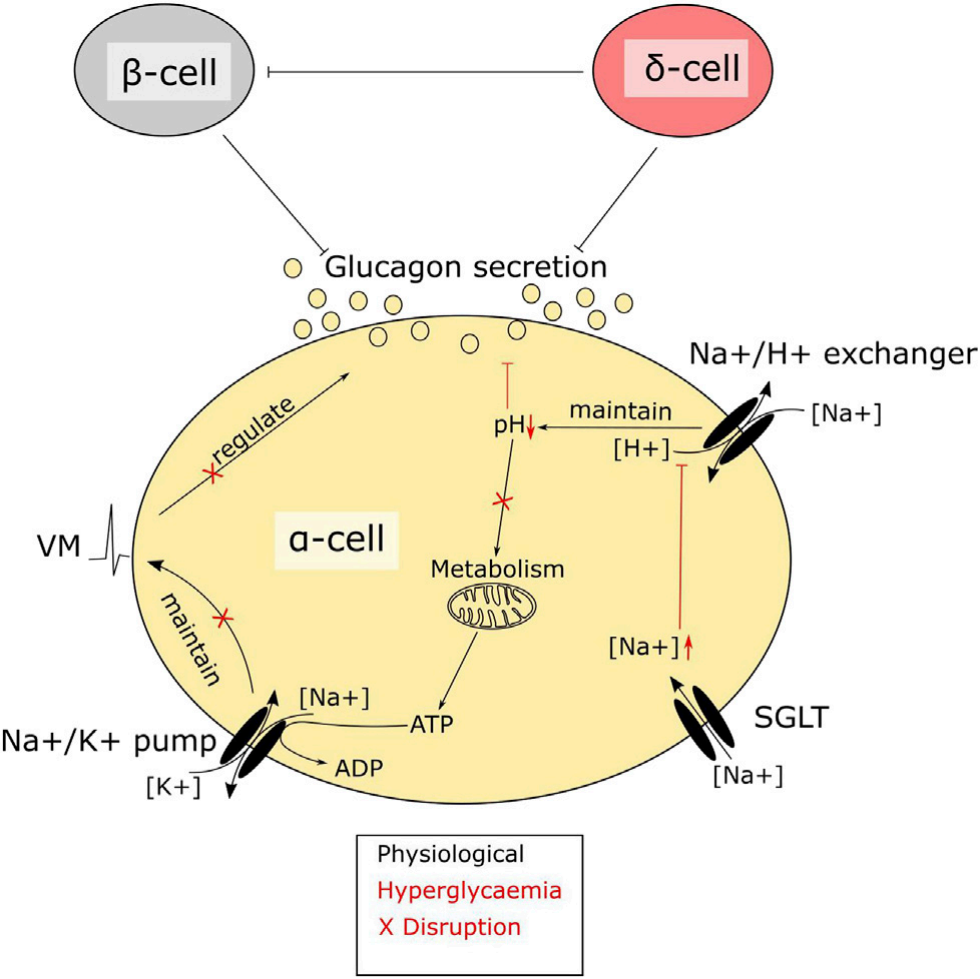
A central role of SGLTs in alpha cell function. SGLTs mediate sodium influx in pancreatic alpha cells. Intracellular sodium levels are tightly linked to alpha cell function. Disruptions due to hyperglycaemia-mediated accumulation of sodium interfere with alpha cell metabolism. The high intracellular sodium affects the function of sodium proton exchangers, resulting in lowering of pH. Maintenance of pH is crucial in alpha cells, as the intrinsic glucose sensing mechanism depends largely on metabolism. Metabolic enzymes, which are sensitive to pH, will–when disrupted–render the cells glucose blind, resulting in dysregulated glucagon secretion.

Alpha cells express not only SGLTs, but also glucose transporter 1 (GLUT1) (Heimberg et al., 1995). GLUT1 is considered the main glucose transporter and is responsible for the majority of glucose transport in alpha cells, Therefore the importance of glucose transport through SGLTs for alpha cell function is unclear. Several findings have suggested that regulation of intracellular sodium levels is important for glucagon secretion (Kalkhoff and Siegesmund, 1981; Bonner et al., 2015; Knudsen et al., 2019) and there seem to be at least three sodium transport systems that are important for alpha cell function: the sodium potassium pump, sodium proton exchangers and SGLTs (Figure 2). In low glucose, the membrane potential is maintained by the sodium potassium pump, which allows alpha cells to be electrically active and secrete glucagon (Briant et al., 2018). Sodium proton exchangers, such as NHE1, maintain intracellular pH and protect against intracellular acidification at higher glucose levels (Knudsen et al., 2019). Given these observations, it could be suggested that it is transport of sodium, rather than glucose, through SGLTs that is important. Few studies have investigated how genetic manipulation of SGLT1 in islets affects glucagon secretion. One such study suggests that in whole body SGLT1 knock out mice on a 60% HFD, glucagon secretion from whole islets was unaltered; however, islets from these mice had an increased proportion of alpha cells, suggesting that secretion may have been impaired. This was recapitulated *in vivo*, where glucose was unable to correctly regulate glucagon secretion during an oral glucose tolerance test, with lower glucagon levels immediately after glucose ingestion and higher glucagon levels after 60 min (Muhlemann et al., 2018). In addition, loss of SGLT1 seems to ameliorate the effects of HFD feeding and streptozotocin on glucose homeostasis (Powell et al., 2013; Muhlemann et al., 2018), a phenotype that aligns with the previous study in SGLT1 KO animals. Other rodent models with impaired glucagon secretion at low glucose, such as the alpha cell HIF1a KO mice, also have lower expression of SGLT1 (Sato et al., 2020). These findings suggest that SGLT1 plays a central role for glucagon secretion in response to glucose and provides a potential explanation for increased circulating glucagon seen in some patients on SGLT2 inhibitors (Ferrannini et al., 2014; Merovci et al., 2014).

### SGLTs in Alpha Cells—A Potential Target for Diabetes Treatment

The beneficial effects of treating hyperglucagonaemia in diabetes is undeniable, and interventions targeting the hormone have already been shown to improve glycaemia in human patients (Kazda et al., 2016). Despite the encouraging results, inhibition of glucagon signalling in diabetic patients has unwanted side effects such as accumulation of fat in the liver (Guzman et al., 2017). Alpha cell dysfunction in T2D is not uniform, although one common trait seems to be impaired secretion at low glucose levels, the response to increased glucose levels seems to fall into two groups; either increased or unchanged secretion (Zhang et al., 2013). This glucagon secretion pattern is similar to islets from T1D patients (Brissova et al., 2018), indicating that alpha cell dysfunction is more general and does not only affect glucose-induced inhibition of glucagon secretion, but also the stimulation of secretion at low glucose. This suggests that improving alpha cell function, rather than limiting hyperglucagonaemia, could be more beneficial for patients.

**FIGURE 3:**
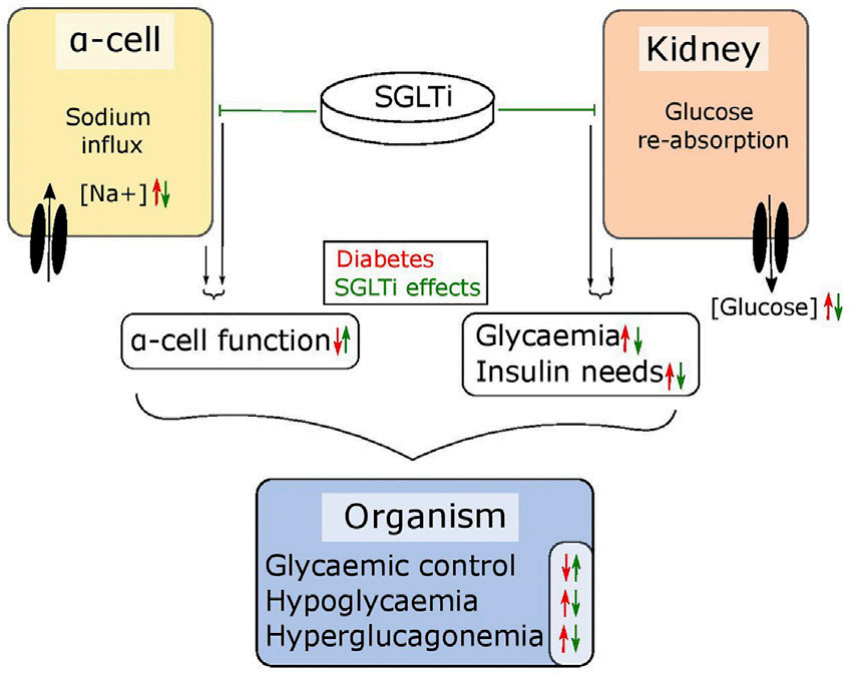
SGLT inhibitors tackle diabetic symptoms from two angles. SGLTs maintain the sodium flux in alpha cells and glucose reabsorption in the kidneys. In diabetic individuals, the sodium influx in alpha cells leads to dysfunctions in glucagon regulation while the reabsorption of glucose in the kidneys further elevates high blood glucose levels. SGLT inhibitors can – depending on their specificity – lower glycaemia by blocking reabsorption in the kidneys, which also lowers the need for required insulin and simultaneously restores alpha cell function. Together, this results in improved glycaemic control.

The abnormal glucagon secretion from alpha cells in diabetes is a consequence of several different impairments in cell function (Zhang et al., 2013; Knudsen et al., 2019; Kellard et al., 2020; Omar-Hmeadi et al., 2020) however, there is a clear metabolic component. Islets from NOD mice and the hyperglycaemic Fumarate hydratase 1 (FH1) beta cell knock out mouse (FH1βKO) have similar patterns of low secretion at low glucose and no inhibition at higher glucose levels (Taborsky et al., 2009; Knudsen et al., 2019). While the reason for the disturbed glucagon section is unclear in NOD mice, βFH1KO mice have impaired substrate oxidation in the alpha cell, a feature they share with the alpha cell specific carnitine palmitoyl transferase 1a (CPT1a) knockout mouse (αCPT1aKO) animals, where substrate oxidation is also compromised and glucagon secretion is reduced at lower glucose levels (Briant et al., 2018). While the effects in αCPT1aKO mice are driven by a direct defect in β-oxidation, the effect in alpha cells from FH1βKO mice may be driven by high extra cellular glucose and increased sodium uptake through SGLT. The higher intracellular sodium is suggested to impair intracellular pH regulation, leading to lower activity of TCA cycle enzymes and lower ATP production (Figure 2). Thus, it could be speculated that the increase in circulating glucagon observed in diabetic patients treated with SGLT2 inhibitors is as reflection of improved alpha cell function and metabolism caused by inhibition of SGLT1, rather than aberrant glucagon secretion. Thus, an increase in fasting glucagon levels would be beneficial for both T1D patients and insulin dependent T2D patients, as both patient groups demonstrate a loss of glucagon secretion in response to hypoglycaemia. Given the available data on SGLT expression and effects of SGLT inhibition in alpha cells, it may be beneficial to explore the effect of inhibitors such as Canagliphlozin, that are more specific for SGLT1, in the treatment of both type 1 and type 2 diabetes (Figure 3). A recent comparative retrospective cohort study assessed the use of different SGLT inhibitors and doses on blood glucose of patients with type 1 diabetes and found reduced insulin needs in treated groups. However, glucagon levels were not quantified in the study and therefore, in this setting, the effect on alpha cell function remains unclear (Palanca et al., 2022). Nonetheless, double-blinded studies using inhibitors targeting SGLT1 over SGLT2 will be needed to fully understand the therapeutic potential. This highlights a potential role of SGLT inhibition in normalising glucose levels. However, as alpha cell function seems to rely on maintaining intracellular sodium levels in specific range, inhibiting SGLT1 may have a limited therapeutic window.

### Coda

We suggest here that sodium plays an important role in the regulation of glucagon secretion. In alpha cells, strict control of intracellular sodium levels is not only required for generating action potentials, but for maintaining plasma membrane potential and intracellular pH. Regardless of the disagreement over the presence of SGLT2 in pancreatic islets, this indicates that SGLT1 may play an important role for alpha cell function. While we still do not understand the role the transporters play in normal alpha cell function, it is clear that inhibition can improve alpha cell function and regulate glucagon secretion in response to hyperglycaemia. Thus, SGLT1 inhibition could have potential as a treatment for alpha cell dysfunction in diabetes.
